# Comparison between Widefield Optical Coherence Tomography Devices in Eyes with High Myopia

**DOI:** 10.3390/diagnostics11040658

**Published:** 2021-04-06

**Authors:** Federico Corvi, Federico Zicarelli, Matteo Airaldi, Salvatore Parrulli, Mariano Cozzi, Davide Monteduro, Francesco Romano, SriniVas R. Sadda, Giovanni Staurenghi

**Affiliations:** 1 Eye Clinic, Department of Biomedical and Clinical Science “Luigi Sacco”, Sacco Hospital, University of Milan, via G.B Grassi 74, 20157 Milan, Italy; federicozicarelli@gmail.com (F.Z.); matteo.airaldi@unimi.it (M.A.); salva.parru90@gmail.com (S.P.); mariano.cozzi88@gmail.com (M.C.); davidemonteduro5@gmail.com (D.M.); francesco.romano@unimi.it (F.R.); giovanni.staurenghi@unimi.it (G.S.); 2 Doheny Eye Institute, University of California at Los Angeles, Los Angeles, CA 90095, USA; ssadda@doheny.org; 3 Department of Ophthalmology, David Geffen School of Medicine at UCLA, Los Angeles, CA 90095, USA

**Keywords:** conjugate image artifact, high myopia, myopia, optical coherence tomography, retina, retrobulbar tissue, sclerochoroidal interface

## Abstract

Background: To compare four different optical coherence tomography (OCT) devices for visualization of retinal and subretinal layers in highly myopic eyes. Methods: In this prospective, observational, cross-sectional study, consecutive patients with high myopia and control subjects were imaged by four OCT devices: Spectralis OCT2, PlexElite 2.0 100 kHz, PlexElite 2.0 200 kHz and the Canon Xephilio OCT-S1. The acquisition protocol for comparison consisted of single vertical and horizontal line scans centered on the fovea. Comparison between the devices in the extent of visible retina, presence of conjugate image or mirror artifacts, visibility of the sclerochoroidal interface and retrobulbar tissue. Results: 30 eyes with high myopia and 30 control subjects were analyzed. The visualized RPE length was significantly different between the OCT devices with Xephilio OCT-S1 imaging the largest extent (*p* < 0.0001). The proportion of eyes with conjugate image artifact was significantly higher with the Spectralis OCT (*p* < 0.0001), and lower with the PlexElite 200 kHz (*p* < 0.0001). No difference in visibility of the sclerochoroidal interface was noted among instruments. The retrobulbar tissue was visible in a higher proportion of eyes using swept-source PlexElite 100 kHz and 200 kHz (*p* < 0.007) compared to the other devices. Conclusions: In highly myopic eyes, the four OCT devices demonstrated significant differences in the extent of the retina imaged, in the prevalence of conjugate image artifact, and in the visualization of the retrobulbar tissue.

## 1. Introduction

Myopia is one of the most common eye problems, and is growing, affecting 1.6 billion individuals worldwide [1]. It has been estimated that the prevalence of myopia and high myopia will increase to 5 billion people and 1 billion people, respectively, by 2050 [1]. Myopia is currently defined by a spherical equivalent of ≤−0.50 diopters or an axial length (AL) > 24.5 mm, while high myopia as a spherical equivalent of ≤−6.00 diopters or an AL of ≥ 26.5 mm [2].

The increase in AL and associated ectasia may lead to structural and functional degenerative changes characteristic of pathologic myopia [3,4,5]. Advances in imaging technologies such as optical coherence tomography (OCT) and three-dimensional magnetic resonance imaging (MRI) have enhanced our understanding of the ocular changes associated with high myopia [3,6]. In fact, the two key factors in the development of pathologic myopia are the elongation of the AL and posterior staphyloma [4]. However, the increase in AL and the posterior staphyloma introduces a substantial challenge to standard clinical ophthalmic imaging techniques. At the same time, the retinal periphery and the posterior pole of high myopic eyes are prone to several complications that require careful assessment and monitoring [7,8]. 

First-generation time-domain OCT devices were able to scan only a restricted portion of the posterior pole, with limited tissue penetration and relatively low resolution [9,10]. Subsequent spectral-domain OCT technology allowed for a more detailed visualization of the posterior pole anatomy and enhanced view of deeper tissues [11,12]. More recently, swept-source OCT has further enhanced penetration of the OCT signal, and with higher scanning speeds, has facilitated the acquisition of larger fields of view [13,14].

The steep curvature of highly myopic eyes can impact the visualization of the posterior segment on OCT. The tilted segments of the scan may impact the visibility of certain retinal structures [15]. The size of the imaging window may also impact how much of the retina can be visualized in a normal orientation without conjugate image artifact impacting the more peripheral aspects of the scan.

Several studies have compared OCT devices with regard to retinal thickness measurements in normal eyes, but thus far there have been no studies comparing the visualization of the retina on OCT in highly myopic eyes among different OCT devices. Given the unique topographical challenges posed by these eyes and the increasing prevalence of myopia, evaluating the performance of current OCT devices would appear to be of importance.

Thus, in this study we compared the visibility of retinal and subretinal structures in highly myopic eyes and normal emmetropic eyes using four different OCT devices.

## 2. Materials and Methods

### 2.1. Study Population

This is a prospective study that adhered to the tenets of the Declaration of Helsinki and was approved by the Luigi Sacco Hospital Ethics Committee in Milan. Written informed consent was obtained from all subjects prior to enrollment. All consecutive highly myopic patients presenting between January 2020 and April 2020 to the Eye Clinic, Department of Biomedical and Clinical Sciences, Luigi Sacco Hospital, University of Milan were offered participation in the study. All who met eligibility criteria accepted and were included. Inclusion criteria were: (1) AL ≥ 26.5 mm and spherical refractive error −6.00 diopters or less and (2) age older than 18 years. Exclusion criteria were: (1) significant media opacity which were thought to preclude the capture of high-quality images by the enrolling physician and (2) poorly cooperative patient as assessed by the OCT operator.

In parallel to the recruitment of these highly myopic subjects, a second cohort of subjects was also enrolled as emmetropic controls with (1) AL < 24.5 mm and spherical refractive error between to +1.0 and −0.50 diopters and (2) age older than 18 years. Exclusion criteria included (1) significant media opacity, (2) any evidence of retinal disease, and (3) poor patient cooperation.

### 2.2. Study Protocol

All subjects underwent a complete ophthalmic evaluation, including assessment of distance best corrected visual acuity, tonometry, slit-lamp biomicroscopy, and indirect fundus ophthalmoscopy. If both eyes met eligibility criteria, one eye was randomly chosen as the study eye. All imaging was performed following pupil dilation at the same visit using four different OCT devices which were selected for this study. The Spectralis OCT2 with enhanced deep imaging (EDI) mode (Spectralis; Heidelberg Engineering, Heidelberg, Germany) was selected as it is a broadly available commercial SD-OCT device that offers customizable scan patterns and various fields of view. The Plex Elite 2.0 100 kHz Dual-Speed (Carl Zeiss Meditec, Inc., Dublin, CA, USA) was selected as it was the first FDA-cleared swept source (SS)—OCT device but offers X/Y scan dimensions roughly similar to the Spectralis OCT2. We also selected the new prototype Plex Elite 2.0 200 kHz Dual-Speed (Carl Zeiss Meditec, Inc, Dublin, California, USA) as it offers similar X and Y dimensions as Spectralis and Plex Elite 2.0 100 kHz but offers the highest Z dimension of all devices. Finally, we included the Canon Xephilio OCT-S1 (Canon, Tokyo, Japan) which is also a swept source OCT device, but offers the largest X and Y dimensions of all of our available devices, albeit not as deep a Z dimension as the Plex Elite 2.0 200 kHz.

This combination of devices was selected to allow us to compare spectral domain vs. swept source technologies as well as different X/Y and Z dimensions with respect to their impact on our ability to assess highly myopic eyes.

### 2.3. Assessment of Axial Length

The AL was measured in the study eye of each patient using the Haag-Streit LENSTAR LS 900. Five consecutive measurements are obtained and averaged to obtain the result. All readings were within 0.02 mm of each other with signal-to-noise ratio of at least 2.0.

### 2.4. Imaging Protocol

All OCT examinations were performed by an expert trained operator (MC). The common scan acquisition for each device included a single horizontal and single vertical line passing through the foveal center, though the precise dimensions and resolution of the scan lines was determined by the capabilities of the specific device (Table 1). Specifically, (1) Spectralis SD-OCT2 with 55° field of view: 17.8 mm × 17.8 mm × 1.9 mm (X by Y by Z) B-scan with 496 transverse pixels along the B-scan or 3.87 μm/pixel; enhanced depth imaging mode and automatic real-time tracking (ART) of 100 (i.e., 100× averaging); (2) Plex Elite 100 kHz HD spotlight with 56° field of view, 16.0 mm × 16.0 mm × 3.0 mm (X by Y by Z) B-scan with 1536 transverse pixels or 1.95 μm/pixel and ART of 100; (3) Plex Elite 200 kHz UHD spotlight with 56° field of view, 16.0 mm × 16.0 mm × 6.0 mm (X by Y by Z) B scan with 3072 transverse pixels or 1.95 μm/pixel and ART of 100; (4) Canon Xephilio OCT-S1 80° by 70° field of view, 23.0 mm × 20.0 mm × 5.3 mm (X by Y by Z) B-scan with 1324 transverse pixels or 4.00 μm/pixel and ART of 100. Images were considered acceptable if the manufacturer specified signal strength was achieved and image was well centered and situated in the fovea. If the scan did not meet these criteria it was repeated.

### 2.5. OCT Analysis

**Figure 1:**
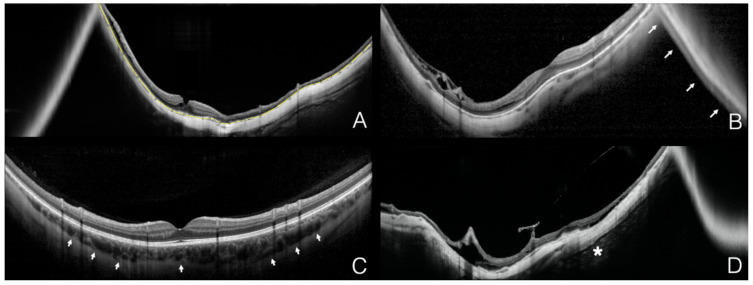
Representative examples of the different qualitative and quantitative parameters analyzed on the optical coherence tomography B-scans. The yellow segmented line (ImageJ measurement tool) delineates the total measurable retinal pigment epithelium in the B-scan (**A**). The white arrows denote the conjugate image artifact (**B**). The white arrowheads delineate the sclerochoroidal interface (**C**). The white asterisk marks the retrobulbar tissue as reflective structures detectable posterior to the sclera (**D**).

Images from different devices were anonymized, assigned a study identifier and presented randomly to the readers (FZ and MA) who graded the scans independently and in a masked fashion. Graders were asked to specifically assess for: (a) presence of conjugate image artifacts (defined as the presence of a segment of flipped or mirror image of the retina), (b) visibility of the sclerochoroidal interface and (c) visibility of retrobulbar tissue (Figure 1). Disagreements between readers were arbitrated by an independent senior reader (GS).

Images were then imported into ImageJ (National Institute of Health, Bethesda, MD, USA) to perform a quantitative analysis of the length of the retinal pigment epithelium (RPE) band visualized in each scan as an approximation of the surface area of retina visualized by each device. The measurement was made in mm after adjusting for the pixel/µm ratio specific to each device. Only the upright segment of retina was included in this measurement and the sections demonstrating conjugate image artifact were excluded.

### 2.6. Statistical Analysis

The statistical analyses were performed using SPSS software v.26.0 (IBM Corp, Armonk, New York, NY, USA). The normality of sample distribution for the quantitative variables was evaluated by means of the Shapiro-Wilk test. The Friedman test was used to compare mean values and then a post hoc test was used to evaluate the differences among instruments. Bland-Altman analysis was also conducted to assess the limits of agreement between the different devices. Qualitative variables were compared using the Chi-square test. Intraclass correlation coefficient (ICCs) and Cohen’s κ were computed to measure the intergrader repeatability for quantitative and qualitative assessments. *p*-Values < 0.05 were considered statistically significant.

## 3. Results

A total of 30 eyes of 30 patients with high myopia were enrolled. The mean age was 52.6 ± 17.5 years, and the cohort included 19 (63%) females and 11 (37%) males. The mean AL was 29.27 ± 1.31 (range 26.95–31.69). A total of 30 eyes of 30 healthy emmetropic controls were also enrolled. The mean age of the controls was 54.5 ± 18.75 years, and the cohort included (57%) females and 13 (43%) males. The mean AL was 24.01 ± 1.38 (range 23.15–24.48).

### 3.1. RPE Length

**Figure 2:**
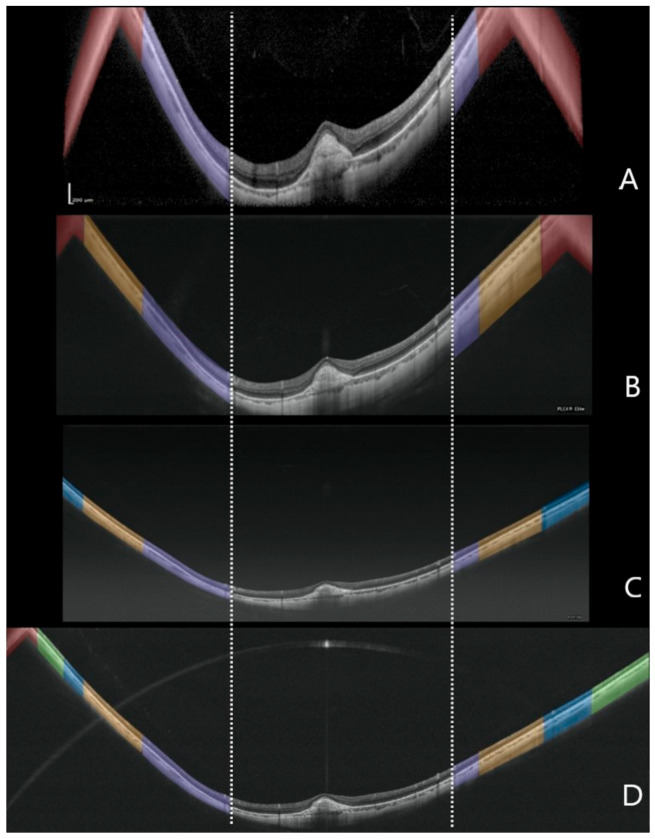
Representative example of a highly myopic eye imaged with four different optical coherence tomography devices. Spectralis OCT2 (**A**); Plex Elite 2.0 100 kHz (**B**); Plex Elite 2.0 200 kHz (**C**) and Canon Xephilio OCT-S1 (**D**). The purple masks show the corresponding zones of visible retina across all the devices. The orange masks indicate the additional corresponding zones of visible retina evident with the Plex Elite 2.0 100 kHz, Plex Elite 2.0 200 kHz and Canon Xephilio OCT-S1. The blue masks show the additional corresponding zones of visible retina evident only on the Plex Elite 2.0 200 kHz and Canon Xephilio OCT-S1. The green masks indicate the additional visible retina only evident with the Canon Xephilio OCT-S1. The red masks show the portion of flipped or mirror image artifact, which is not included in the quantitative measurements.

In the high myopia cohort, the RPE length measured on the horizontal and vertical scans did not follow a normal distribution (*p* < 0.0001) and the mean values for each device are reported in Table 2. The four OCT measured different mean values for the RPE length in the horizontal and vertical scans (*p* < 0.0001). The RPE length measured by the Xephilio OCT-S1 was significantly greater than the other devices (*p* < 0.0001) followed by the Plex Elite 200 kHz, Plex Elite 100 kHz, and finally the Spectralis OCT2, which measured a smaller RPE length than all of the other devices (*p* < 0.0001) (Figure 2).

**Figure 3:**
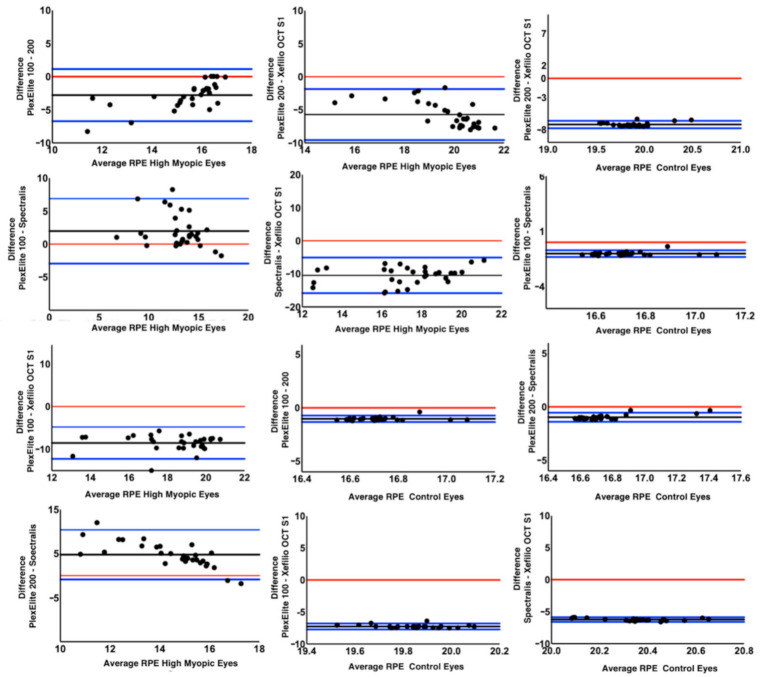
Bland-Altman plots comparing the amount of visible retinal pigment epithelium between the different optical coherence tomography devices for the horizontal scans in high myopia cohort and controls. The black lines indicate the mean difference between the different devices. The blue lines show the upper and lower limits of agreements. The red line denotes the zero difference between the devices.

**Figure 4:**
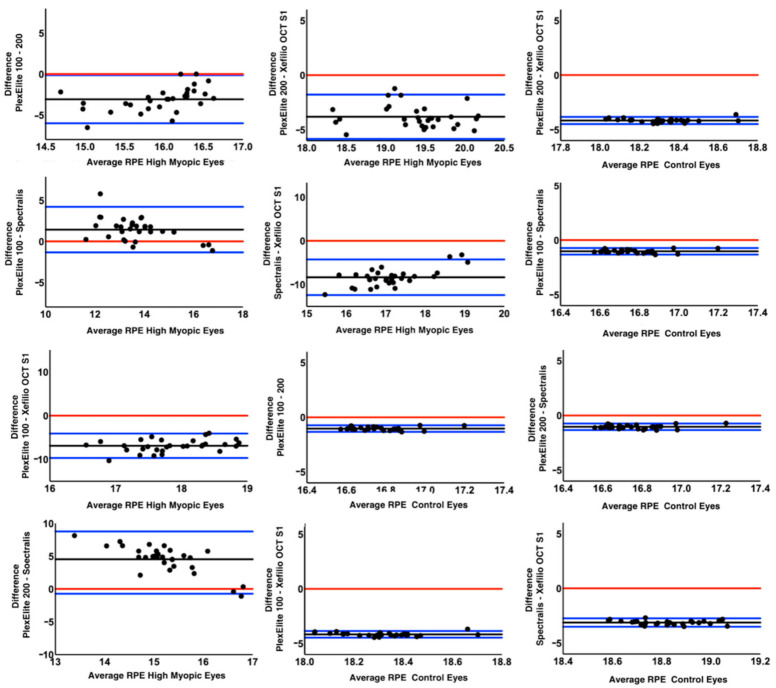
Bland-Altman plots comparing the amount of visible retinal pigment epithelium between the different optical coherence tomography devices for the vertical scans in high myopia cohort and controls emmetropic eyes. The black lines indicate the mean difference between the different devices. The blue lines show the upper and lower limits of agreements. The red line denotes the zero difference between the devices.

For control eyes the mean RPE length in both the horizontal and vertical scans was also significantly different among the four OCT devices (*p* < 0.0001). Xephilio OCT-S1 again imaged more RPE compared to the other devices (*p* < 0.0001), followed by Spectralis SD-OCT2 while no difference was observed between Plex Elite 100 kHz and Plex Eite 200 kHz (*p* = 0.194 and *p* = 0.44 for the horizontal and the vertical scan). In particular, both Plex Elite devices displayed a smaller RPE length compared to the other devices. Bland-Altman analysis of the 12 possible pairwise comparisons for the horizontal and vertical scans among the four devices was used to investigate the limits of agreements (Figure 3 and Figure 4). Intergrader agreement for RPE measurements was high with ICC of 0.992 (95% CI 0.982–0.996).

### 3.2. Conjugate Image Artifact

In the high myopia cohort, the proportion of eyes with conjugate image artifact (CIA) was significantly different among the four OCT devices (*p* < 0.0001) (Table 2). Specifically, the Spectralis SD-OCT2 showed a significantly higher proportion of eyes with CIA compared to the other devices (*p* < 0.0001) followed by the Plex Elite 100 kHz, Xephilio OCT-S1 and Plex Elite 200, which showed a lower proportion (*p* < 0.0001) (Table S1).

In the control group, no differences were observed among the devices (*p* = 0.4). Cohen’s kappa coefficient was 0.94 between graders for this assessment.

### 3.3. Sclerochoroidal Interface Visibility

No differences were found among the four OCT devices with respect to the visibility of the sclerochoroidal interface in both groups (*p* = 1) (Table 2 and Table S1). Inter-grader agreement was perfect with a Cohen’s kappa coefficient of 1.

### 3.4. Retrobulbar Tissue Visibility

In the high myopia cohort, the visibility of the retrobulbar tissue was significantly different between the technologies (*p* < 0.0001), with the retrobulbar tissue visualized in a higher proportion of eyes imaged by the Plex Elite 100 kHz and Plex Elite 200 kHz (*p* < 0.007). No differences were observed between the Plex Elite 100 kHz and Plex Elite 200 kHz (*p* = 0.51) and the Xephilio OCT-S1 vs. Spectralis SD-OCT2 (*p* = 0.12) (Table 2 and Table S1).

In contrast, when considering the group of control eyes, the visibility of the retrobulbar tissue was not significantly different among the four OCT devices (*p* = 0.57). Cohen’s kappa coefficient was 0.94 for agreement between graders.

## 4. Discussion

**Figure 5:**
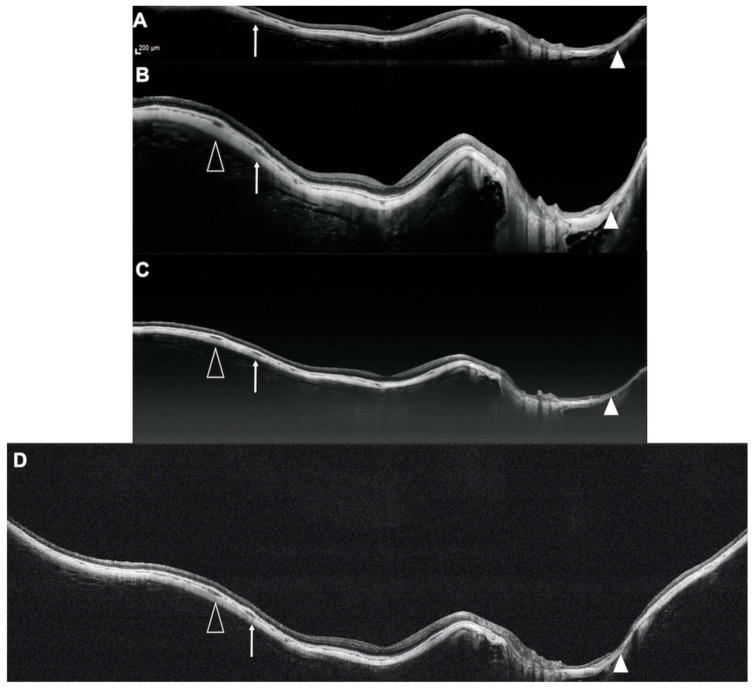
Representative example of a highly myopic eye imaged with four different optical coherence tomography devices. Spectralis OCT2 (**A**); Plex Elite 2.0 100 kHz (**B**); Plex Elite 2.0 200 kHz (**C**) and Canon Xephilio OCT-S1 (**D**). The arrowheads, the white arrowheads and the white arrows indicate the same structures in the different B-scans highlighting the different extent of retina visualized by the different devices.

In this study, we compared four different OCT devices for imaging highly myopic and normal emmetropic controls with regards to the length of measurable RPE, prevalence of conjugate image artifacts, and visibility of the retrobulbar tissue (Figure 5), and we observed significant differences among devices.

Notably, the RPE measurement was significantly different among the four instruments, which was most clearly evident in the Bland-Altman analysis (Figure 3 and Figure 4). The Xephilio OCT-S1, which has the longest X dimension, not surprisingly, demonstrated the longest RPE measurement on both horizontal and vertical scans, regardless of the AL. In contrast, the Spectralis SD-OCT2 imaged less RPE in both horizontal and vertical scans compared to the other OCT devices, in the highly myopic eyes. However, in the control group, the Plex Elite 100 kHz imaged the least RPE, and the RPE measured was similar to the Plex Elite 200 kHz. We suspect these differences among devices and between myopic and emmetropic eyes can be explained directly by the dimensions of the scan window. While the Spectralis had the smallest Z dimension of any of the devices, the Spectralis had a longer X and Y dimension compared to the Plex Elite (100 kHz or 200 kHz). Notably, the Xephilio OCT-S1 had the largest X and Y dimension, and a Z dimension that was greater than all the devices except for the PlexElite 200 kHz. Taken together, our findings suggest that the X and Y dimension are most influential with regards to the visualization of the retinal/RPE suface in non-myopic eyes, whereas the Z dimension is most important in highly myopic eyes. These findings make sense when one considers the ectasia and steep curvature present in highly myopic eyes.

The conjugate image or mirror artifact represents an intrinsic limit of the Fourier transformation used in both spectral domain and swept-source OCTs, and it occurs when the structure visualized in the B-scan extends beyond the Z dimension window of the particular OCT device [16]. This artifact is important as generally only the upright (“non-flipped”) portion of the retina is clearly evaluable and thus dictates the effective limits of the scan even if the X or Y dimensions are significantly longer. Our findings also confirm that a longer AL is an important determinant of the presence of these conjugate image artifacts.

Even among the emmetropic eyes, the two devices with the shortest Z-scan dimension (Spectralis SD-OCT2 and Plex Elite 100 kHz) demonstrated a few eyes with conjugate image artifact, which was uncommon in the Xephilio OCT-S1 and Plex Elite 200 kHz scans. However, in the highly myopic eyes (AL ≥ 26.5 mm), the proportion of eyes with CIA increased for all the devices. Notably, the Xephilio OCT-S1 showed a significantly higher proportion of eyes with CIA compared to the Plex Elite 200 kHz despite a difference in the Z-scan dimension of only 0.7 mm. Despite the higher proportion of mirror/CIA artifacts, as noted above, the Xephilio OCT-S1 still demonstrated the most visible retina as reflected in the measured RPE length.

The ability to visualize signals in the retrobulbar tissue beyond the sclera is known to be dependent on the wavelength used by the OCT technology [13,17,18,19,20]. In this study, no differences were observed in the group of control eyes where the choroidal thickness is expected to be thicker compared to myopic eyes, which may reduce the chance to image even deeper structures. In contrast, in the highly myopic eyes, the PlexElite 100 kHz and 200 kHz showed better signal penetration and visualization of the deep structures. This finding may reflect the properties of the light source and the signal roll-off characteristics of the device and to different resolution.

This study has several limitations to be considered. First, the RPE length was measured on two orthogonal B-scans passing through the foveal center. Although these were good-fixating patients, there may be some variation in the exact position of the B-scans among devices, as the scans were not registered to each other across devices. The B-scans were acquired positioning the retina in the lower part of the acquisition window in order to maximize the amount of RPE visible. This could have reduced the visualization of the retrobulbar tissue in the SS-OCT devices due to sensitivity roll-off from the zero-delay line. Finally, while the RPE length was quantitative, the remaining parameters were subjective, and thus potentially subject to inter-observer variability. However, to mitigate this we used two masked graders and demonstrated a high level of repeatability between graders.

In summary, in this study we compared images acquired by four different OCT devices in eyes highly myopia and control emmetropic eyes, and demonstrated significant differences in the amount of the retina correctly displayed, in the prevalence of mirror artifacts, and in the visibility of retrobulbar tissue. While the X and Y dimensions were the most important predictors for the extent of retina visualized in non-highly myopic eyes, the Z dimension was of greatest importance in myopic eyes. Our findings may be of value in defining the optimal device and acquisition parameters for future studies of highly myopic eyes.

## Figures and Tables

**Table 1:** Characteristics of optical coherence tomography devices.

Device	Version	Wavelength	Technology	Scan Speed	Field of View	X, Y, Z Scan Dimension	Z Scan Pixels µm/pixel
Spectralis SD-OCT2	1.10.4.0	880 µm	Spectral domain	85,000 A-scan/s	55°	17.8 mm, 17.8 mm, 1.9 mm	496 pixel 3.87 µm/pixel
Plex Elite 100 kHz	2.0.1.47652	1060 µm	Swept source	100,000 A-scan/s	56°	16 mm, 16 mm, 3 mm	1536 pixel 1.95 µm/pixel
Plex Elite 200 kHz	2.0.1.47652	1060 µm	Swept source	100,000 A-scan/s	56°	16 mm, 16 mm, 6 mm	3072 pixel 1.95 µm/pixel
Xephilio OCT-S1	1.0.0.18	1060 µm	Swept source	100,000 A-scan/s	80° by 70°	23 mm, 20 mm, 5.3 mm	1324 pixel 4 µm/pixel

**Table 2:** OCT findings in high myopia cohort and emmetropic controls.

Features	Spectralis SD-OCT 2	Plex Elite 100 kHz	Plex Elite 200 kHz	Xephilio OCT-S1
RPE length horizontal scan, mm				
High Myopia	12.05	14.01	16.81	22.52
Controls	17.16	16.02	16.25	23.46
RPE length vertical scan, mm				
High Myopia	12.96	14.4	17.58	21.29
Controls	17.13	16.2	16.26	20.49
Conjugate Image Artifact, *n* (%)				
High Myopia	61 (51)	54 (45)	6 (5)	39 (33)
Controls	8 (6)	4 (3)	1 (0)	1 (0)
Sclerochoroidal interface, *n* (%)				
High Myopia	120 (100)	120 (100)	120 (100)	120 (100)
Controls	120 (100)	120 (100)	120 (100)	120 (100)
Retrobulbar tissue visibility, *n* (%)				
High Myopia	21 (18)	53 (44)	47 (39)	32 (27)
Controls	0 (0)	3 (3)	3 (3)	1 (1)

RPE, retinal pigment epithelium.

## Data Availability

Information is available in the article.

## Supplementary Materials

The following are available online at https://www.mdpi.com/article/10.3390/diagnostics11040658/s1, Table S1. OCT features. Pairwise comparisons between instruments.
