# Supplementary material for: Comparison between Widefield Optical Coherence Tomography Devices in Eyes with High Myopia
Source: Diagnostics (Basel). 2021 Apr 6;11(4):658. doi: 10.3390/diagnostics11040658 (PMC8067388; doi:10.3390/diagnostics11040658)
Supplement: Supplementary file 1 [file diagnostics-11-00658-s001.pdf]

Table S1. OCT features. Pairwise comparisons between instruments.

Conjugate Image Artifact			
**OCT Comparison**		**Group**	***p* Value**
Plex Elite 100 kHz	Plex Elite 200 kHz	Entire cohort	<0.0001
Plex Elite 100 kHz	Spectralis SD-OCT2	Entire cohort	1.00
Plex Elite 100 kHz	Xephilio OCT-S1	Entire cohort	0.035
Plex Elite 200 kHz	Spectralis SD-OCT2	Entire cohort	<0.0001
Plex Elite 200 kHz	Xephilio OCT-S1	Entire cohort	<0.0001
Spectralis SD-OCT2	Xephilio OCT-S1	Entire cohort	0.005
Plex Elite 100 kHz	Plex Elite 200 kHz	AXL < 26.5 mm	1.000
Plex Elite 100 kHz	Spectralis SD-OCT2	AXL < 26.5 mm	1.000
Plex Elite 100 kHz	Xephilio OCT-S1	AXL < 26.5 mm	1.000
Plex Elite 200 kHz	Spectralis SD-OCT2	AXL < 26.5 mm	0.188
Plex Elite 200 kHz	Xephilio OCT-S1	AXL < 26.5 mm	1.000
Spectralis SD-OCT2	Xephilio OCT-S1	AXL < 26.5 mm	0.215
Plex Elite 100 kHz	Plex Elite 200 kHz	AXL > 26.5 mm	<0.0001
Plex Elite 100 kHz	Spectralis SD-OCT2	AXL > 26.5 mm	0.04
Plex Elite 100 kHz	Xephilio OCT-S1	AXL > 26.5 mm	0.01
Plex Elite 200 kHz	Spectralis SD-OCT2	AXL > 26.5 mm	<0.0001
Plex Elite 200 kHz	Xephilio OCT-S1	AXL > 26.5 mm	<0.0001
Spectralis SD-OCT2	Xephilio OCT-S1	AXL > 26.5 mm	0.0003
**Sclerochoroidal Interface**			
**OCT Comparison**		**Group**	***p* Value**
Plex Elite 100 kHz	Plex Elite 200 kHz	Entire cohort	1.000
Plex Elite 100 kHz	Spectralis SD-OCT2	Entire cohort	1.000
Plex Elite 100 kHz	Xephilio OCT-S1	Entire cohort	1.000
Plex Elite 200 kHz	Spectralis SD-OCT2	Entire cohort	1.000
Plex Elite 200 kHz	Xephilio OCT-S1	Entire cohort	1.000
Spectralis SD-OCT2	Xephilio OCT-S1	Entire cohort	1.000
Plex Elite 100 kHz	Plex Elite 200 kHz	AXL < 26.5 mm	1.000
Plex Elite 100 kHz	Spectralis SD-OCT2	AXL < 26.5 mm	1.000
Plex Elite 100 kHz	Xephilio OCT-S1	AXL < 26.5 mm	1.000
Plex Elite 200 kHz	Spectralis SD-OCT2	AXL < 26.5 mm	1.000
Plex Elite 200 kHz	Xephilio OCT-S1	AXL < 26.5 mm	1.000
Spectralis SD-OCT2	Xephilio OCT-S1	AXL < 26.5 mm	1.000
Plex Elite 100 kHz	Plex Elite 200 kHz	AXL > 26.5 mm	1.000
Plex Elite 100 kHz	Spectralis SD-OCT2	AXL > 26.5 mm	1.000
Plex Elite 100 kHz	Xephilio OCT-S1	AXL > 26.5 mm	1.000
Plex Elite 200 kHz	Spectralis SD-OCT2	AXL > 26.5 mm	1.000
Plex Elite 200 kHz	Xephilio OCT-S1	AXL > 26.5 mm	1.000
Spectralis SD-OCT2	Xephilio OCT-S1	AXL > 26.5 mm	1.000
**Retrobulbar tissue visibility**			
**OCT Comparison**		**Group**	***p* Value**
Plex Elite 100 kHz	Plex Elite 200 kHz	Entire cohort	0.0002
Plex Elite 100 kHz	Spectralis SD-OCT2	Entire cohort	<0.0001
Plex Elite 100 kHz	Xephilio OCT-S1	Entire cohort	<0.0001
Plex Elite 200 kHz	Spectralis SD-OCT2	Entire cohort	0.162
Plex Elite 200 kHz	Xephilio OCT-S1	Entire cohort	0.395
Spectralis SD-OCT2	Xephilio OCT-S1	Entire cohort	0.661
Plex Elite 100 kHz	Plex Elite 200 kHz	AXL < 26.5 mm	1.000

Plex Elite 100 kHz	Spectralis SD-OCT2	AXL < 26.5 mm	1.000
Plex Elite 100 kHz	Xephilio OCT-S1	AXL < 26.5 mm	1.000
Plex Elite 200 kHz	Spectralis SD-OCT2	AXL < 26.5 mm	1.000
Plex Elite 200 kHz	Xephilio OCT-S1	AXL < 26.5 mm	1.000
Spectralis SD-OCT2	Xephilio OCT-S1	AXL < 26.5 mm	0.46
Plex Elite 100 kHz	Plex Elite 200 kHz	AXL > 26.5 mm	0.51
Plex Elite 100 kHz	Spectralis SD-OCT2	AXL > 26.5 mm	<0.0001
Plex Elite 100 kHz	Xephilio OCT-S1	AXL > 26.5 mm	<0.0001
Plex Elite 200 kHz	Spectralis SD-OCT2	AXL > 26.5 mm	<0.0001
Plex Elite 200 kHz	Xephilio OCT-S1	AXL > 26.5 mm	<0.0001
Spectralis SD-OCT2	Xephilio OCT-S1	AXL > 26.5 mm	0.12
